# Seasonality of water quality and diarrheal disease counts in urban and rural settings in south India

**DOI:** 10.1038/srep20521

**Published:** 2016-02-12

**Authors:** Alexandra V. Kulinkina, Venkat R. Mohan, Mark R. Francis, Deepthi Kattula, Rajiv Sarkar, Jeanine D. Plummer, Honorine Ward, Gagandeep Kang, Vinohar Balraj, Elena N. Naumova

**Affiliations:** 1 Department of Civil and Environmental Engineering, Tufts University, Medford, MA, USA; 2 Department of Community Health, Christian Medical College, Vellore, Tamil Nadu, India; 3 Division of Gastrointestinal Sciences, Christian Medical College, Vellore, Tamil Nadu, India; 4 Department of Civil and Environmental Engineering, Worcester Polytechnic Institute, Worcester, MA, USA; 5 Division of Geographic Medicine and Infectious Diseases, Tufts Medical Center, Boston, MA, USA

## Abstract

The study examined relationships among meteorological parameters, water quality and diarrheal disease counts in two urban and three rural sites in Tamil Nadu, India. Disease surveillance was conducted between August 2010 and March 2012; concurrently water samples from street-level taps in piped distribution systems and from household storage containers were tested for pH, nitrate, total dissolved solids, and total and fecal coliforms. Methodological advances in data collection (concurrent prospective disease surveillance and environmental monitoring) and analysis (preserving temporality within the data through time series analysis) were used to quantify independent effects of meteorological conditions and water quality on diarrheal risk. The utility of a local calendar in communicating seasonality is also presented. Piped distribution systems in the study area showed high seasonal fluctuations in water quality. Higher ambient temperature decreased and higher rainfall increased diarrheal risk with temperature being the predominant factor in urban and rainfall in rural sites. Associations with microbial contamination were inconsistent; however, disease risk in the urban sites increased with higher median household total coliform concentrations. Understanding seasonal patterns in health outcomes and their temporal links to environmental exposures may lead to improvements in prospective environmental and disease surveillance tailored to addressing public health problems.

India holds about 16% of the world’s population and only 4% of its fresh water resources. Precipitation patterns exhibit high temporal variability with nearly 80% of the annual rainfall occurring in the monsoon season. Spatial variability in rainfall contributes to periodic floods and droughts in different parts of the country^1^. Overall, with an estimated annual per capita water availability of 1,588 m^3^ in 2010, a decrease from 1,816 m^3^ in 2001^2^, India is currently classified as water stressed. Water stress is defined as annual water availability of less than 1700 m^3^ per person, whereas water scarcity occurs when it drops below 1000 m^3^ 3.

One of the major contributing factors to India’s water problem is unsustainable groundwater management. Over 60% of agricultural and 85% of domestic water demands are met through groundwater^4^. A dramatic increase in private withdrawals for irrigation since the 1960s has resulted in a significant drop in the groundwater table in many areas of the country, including Tamil Nadu, and has contributed to deterioration in groundwater quality^5^,^6^. At the current rates of population growth and urbanization, the country is projected to continue to struggle with water quantity and quality issues in the future^1^.

In 2008, WHO-UNICEF estimated that 96% of the urban and 84% of the rural population in India had access to improved water. However, access inferred by these high percentages does not constitute adequate quality and equitable distribution^3^. Many Indian cities, for example, receive water for only a few hours per day necessitating storage, which is associated with water quality (WQ) deterioration. Additionally, water is often already contaminated at the point of gathering because of aged pipelines running adjacent to open drainage channels in highly contaminated environments by practices such as open defecation^7^. In this context, an estimated 37.7 million Indians are affected by waterborne diseases annually with 1.5 million diarrheal deaths in children^3^.

Water quality^8^,^9^,^10^ and diarrheal infections^11^,^12^,^13^,^14^,^15^,^16^,^17^ have been shown to exhibit seasonality. Associations between WQ and diarrhea are difficult to ascertain due to multiple exposure pathways and weak associations between current microbiological WQ indicators and disease causing organisms^18^, which may also vary seasonally^19^. As a result, associations between indicator bacteria in drinking water and disease risk are largely inconclusive^20^,^21^. Associations between diarrheal infections and meteorological parameters such as rainfall and temperature are not uniform across climates zones and time periods, highlighting a complex relationship between weather, WQ and waterborne diseases^12^,^17^.

The present study examined the seasonal patterns of self-reported diarrheal disease counts and WQ in public (i.e. street level public taps in a groundwater fed distribution system) and private (i.e. household water storage containers) domains in urban and rural sites in Vellore District, Tamil Nadu, India (Fig. 1). All study parameters were measured prospectively at semi-regular intervals allowing for establishing temporal relationships between the exposure (WQ) and outcome (diarrheal cases). The data were analyzed using regression models applied to time series data considering the effects of meteorological parameters and the use of the local Tamil calendar.

## Methods

### Study area: population and drinking water supplies

**Figure 1:**
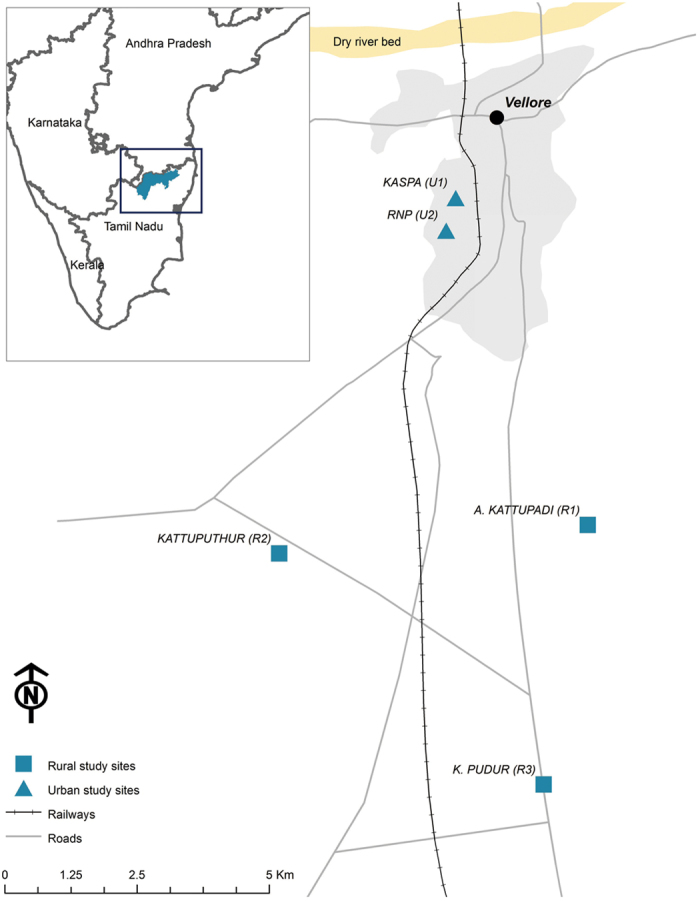
Map of the study area. The meteorological station is located in Vellore town. The figure was created by A. Kulinkina in ArcGIS software (version 10.2.2) using data layers from ML Infomap, 124-A Katwaria Sarai, New Delhi 110 016.

The study was conducted between August 2010 and March 2012 in two urban and three rural sites in Vellore District, Tamil Nadu, India (Fig. 1). The urban sites, Kaspa (U1) and RNP (U2), are geographically adjacent semi-urban slums located on the western outskirts of Vellore town, with majority of the residents earning their wages through unskilled labor. The rural sites, A. Kattupadi (R1), Kattuputhur (R2) and K. Pudur (R3), are villages located 5–10 km south of Vellore town with agricultural labor serving as the predominant source of income.

The U1 and U2 sites share a common groundwater source, a well near a dry river bed approximately 5 km north of Vellore town. Upon withdrawal, water is provided to U1 and U2 through a public tap system managed by the Vellore Municipal Corporation (VMC). Because VMC supplies other nearby communities from the same source on a rotational schedule, the frequency of water provision to U1 and U2 ranges from once every 2 days to once every 28 days depending on seasonal water availability in the aquifer. Water is treated and chlorinated according to the VMC operators. However, chlorination was found to be irregular and unreliable in Vellore^22^,^23^ and in other Indian water systems^7^. R1, R2 and R3 rely on similar public piped water systems but water is drawn from their own source wells nearby (i.e. rural sites do not share a water source), making water available for several hours almost every morning.

In both urban and rural sites, because water provision is intermittent with water flowing for a few hours at a time, positive pressure is not maintained in the piped systems making them susceptible to fecal contamination^24^. In addition to the piped systems, daily water needs in all communities are supplemented by a few remaining manual hand pumps, Rajiv Gandhi mechanized wells, and in the urban sites by tanker trucks provided by the VMC during especially dry conditions. These additional water sources were not considered in the present study.

### Recruitment, data collection and processing

The study was approved by the Institutional Review Boards (IRB) at Christian Medical College (CMC), Vellore, India and Tufts University School of Medicine, Boston, USA. All study activities were carried out in accordance with the approved guidelines. Prior to recruitment, the study area was enumerated by trained study staff in a door-to-door survey (census) identifying 877 eligible households (i.e. with children aged <5 years). Three hundred families (160 out of 719 eligible urban and 140 out of 158 eligible rural families) were recruited and enrolled between August 2010 and March 2011 using staggered enrollment and followed for 12 months. Out of 300 families, 279 (146 urban and 133 rural) completed the study. The study lasted for a total of 86 weeks, with most study weeks (weeks 29 through 79) containing at least 276 households under observation^25^. Over the follow up time, during each week of the study the proportion of children under 5 was steady at 25%. Written informed consent was obtained from the head of the household, verbal consent from all members of the family, and assent from the children.

Trained field workers carried out weekly household surveillance of diarrheal episodes in the study families. Standard case definition for diarrhea, having three or more loose watery stool occurring over a 24-hour period^26^, was used. During a household visit, information on diarrhea was collected for each day since the last visit and was recorded in the diarrheal surveillance form. Additionally, the study families were provided with contact numbers of the field staff and were encouraged to report a diarrheal episode in any family member. The primary care giver or other adult female family members provided information about the children.

Water samples were collected in two domains: public domain, defined as public street level taps in the distribution systems, and private domain, represented by water stored in containers in the households. The public domain was sampled between January 2011 and March 2012 (weeks 23 through 85 of the study) from a randomly chosen subset of all available public taps (Table 1) following a semi-structured collection scheme. Each study tap was sampled approximately once per month with an average of 7 samples per week collected in the urban and 13 in the rural sites. The sampling scheme was influenced by water availability in the piped system, travel convenience and the processing speed of the laboratory facilities. The private domain was sampled between December 2010 and March 2012 (weeks 19 through 85 of the study). Each study household was sampled approximately four times per year with an average of 13 samples per week collected in the urban and 11 in the rural sites. The total numbers of public and private domain samples were 1,062 and 1,218, respectively.

Water samples from both domains were analyzed for the following common water quality (WQ) parameters: pH, chlorine residual (not detected in any of the samples and not considered further in the analysis), nitrate (NO_3_^−^, ppm) and total dissolved solids (TDS, ppm) using standard testing kits (HiMedia Labs Pvt. Ltd.). Total coliforms (TC, CFU/100 mL) and fecal coliforms (FC, CFU/100 mL) were quantified using MacConkey and M-FC media, respectively (HiMedia Labs Pvt. Ltd.). Analyses were conducted by the Wellcome Research Laboratory at CMC. Three dilutions were initially performed for TC (direct, 1:10, and 1:100) and two for FC (direct and 1:10) using a single sample for each dilution. Upon observation of high concentrations of TC in most samples in the initial month of testing (recorded as TNTC, too numerous to count), only the 1:100 dilution was continued for TC throughout the study.

TC and FC concentrations were calculated by averaging either all dilution plates that resulted in the ideal range or in the enumerable range if no dilutions in the ideal range were available for the sample. The ideal range was defined as 20–80 or 20–60 colonies per plate for TC and FC, respectively^27^. The enumerable range was defined as 0–300 colonies per plate for both TC and FC as the limits of quantification per laboratory operating procedures. Samples resulting in all TNTC dilutions were replaced with a value of twice the upper limit of the countable range of the highest dilution^28^: 12 (1.1%) TC and 18 (1.7%) FC measurements in the public domain; 86 (7.1%) TC and 82 (6.7%) FC measurements in the private domain. Samples that resulted in non-detect observations for all dilutions were replaced with 1 colony (limit of detection) prior to dilution adjustment^29^: 10 (0.9%) TC and 43 (4.0%) FC measurements in the public domain; 2 (0.2%) TC and 49 (4.0%) FC measurements in the private domain.

Daily meteorological records for ambient temperature (°C) and rainfall (mm) were obtained from the India Meteorological Department from a monitoring station located in Vellore town (Fig. 1). Data obtained from the Vellore station were applied to describe weather in all study sites, which are located within a 10-km radius of the station.

### Data analysis

We examined the seasonal patterns in WQ and diarrheal disease counts by first creating weekly time series of all study parameters for urban and rural sites. All collected measurements contained a date associated with each observation (i.e. date of a diarrheal event onset, date a water sample was collected, date of meteorological observation). Based on the date, study week was assigned to each observation starting from week 1 through week 86. Diarrheal disease surveillance data and meteorological observations were available for all 86 study weeks. WQ was measured during weeks 23–85 in the public and 19–85 in the private domain with some of the study weeks containing no measurements. Depending on the type of model and whether the variable was used as a predictor or an outcome, either raw or aggregated values (e.g. cumulative diarrheal episode counts, median WQ values) were used in the analysis.

Sequential model building approach was applied using R statistical software (version 3.1.0). For exploratory analysis, diarrheal disease counts, meteorological and WQ parameters were compared across six two-month long seasons according to a locally used Tamil calendar (Table 2). Based on the outcome of interest, two types of regression models were developed: generalized linear model (GLM) with a Gaussian distribution for WQ parameters (denoted as A); and a GLM with a Poisson distribution for weekly aggregated diarrheal disease counts (denoted as B) (Table 3). To ensure linear relationships with the outcome of interest, rainfall, TC and FC were log_10_ transformed. Based on the research question, the model development progressed from a description of seasonal patterns in the outcome variable by using the Tamil calendar (Model 1) to a model with two harmonic terms to account for the possibility of multiple peaks (Model 2), and to a model with harmonic terms adjusted for temperature and rainfall (Model 3). Model 4 examined the effects of private domain WQ on diarrheal disease counts, adjusting for seasonal harmonics, temperature and rainfall. All models also accounted for trend in the outcome variable.

Models for WQ parameters as outcome variables were conducted separately for each parameter, for public and private domains, and for urban and rural sites. All models for cumulative weekly diarrheal disease counts were conducted separately for urban and rural sites and for both sites combined and were adjusted for the number of people under observation for a given week using an offset. Relative risk (RR) and the 95% confidence interval (CI) for diarrheal disease counts attributed to trend, meteorological parameters and water quality parameters were estimated. RR associated with the trend variable in the diarrheal disease count models reflects the predicted change in diarrheal risk over 52 weeks.

All models were tested to ensure that potential effects associated with collinearity, autocorrelation, overdispersion, lag, and missing data are taken into account. To address colliniarity effects, associations across weekly WQ and meteorological parameters were examined using Spearman correlations (Supplemental Material, Table S1). The temporal serial correlations among parameters and with outcomes were assessed to determine the lag structure using autocorrelation function (ACF) plots. A 3-week lag for rainfall exhibited the maximum serial correlation with diarrheal disease counts; thus, Model 3 for diarrheal disease counts was conducted with meteorological data with no temporal lags and for rainfall with a 3-week lag. Interaction between temperature and rainfall variables was also explored and omitted from the models for the ease of interpretation due to its marginal effect. The quality of model fit was assessed based on the percent of variability explained by the model, calculated from the null and residual deviances, Q = (null deviance–residual deviance)/null deviance*100% and AIC (data not shown).

To explore the effects of missing WQ values, as a sensitivity analysis, two versions of Model 4 were conducted. Version 1 utilized only weeks during which private domain WQ values were available (33 in urban and 35 in rural sites). Version 2 used time series where missing WQ values were imputed using linear interpolation based on two adjacent measurements if available (56 in urban and 58 in rural sites). The lag structure between diarrheal disease counts and imputed WQ values was also explored. One week lags for TC and FC concentrations were considered, but did not yield any improvements in the models and the associations maintained their magnitude and direction. All models were tested for autocorrelation in the residuals; none demonstrated significant autocorrelation.

## Results

### Temporal patterns in water quality

Based on the Indian drinking water standards^5^, microbiological contamination is a priority WQ concern in the study area. Median (SD) TC and FC concentrations were approximately 2,700 (7,500) and 80 (830) CFU/100 mL respectively in the public domain samples, and 4,900 (15,000) and 220 (1,500) CFU/100 mL in the private domain samples. Nearly all samples (99–100% depending on site and domain) exceeded the 50 CFU/100 mL TC standard for class A drinking water. Most of the samples (87% in rural and 91% in urban public domain; 90% in rural and 92% in urban private domain) also had >10 FC colonies per 100 mL. Chlorine residual was not detected in any of the samples. pH was generally in the desirable range (6.5 and 8.5), with ~5% of the household and 2% of the tap water samples exceeding 8.5. All of the samples were below the 45 mg/L standard for nitrate. Nearly all samples exceeded the desirable 500 mg/L TDS concentration (98.5%) and about 5% were above 1,000 mg/L.

In defining the seasons based on the Tamil calendar, we determined that TS3 and TS5 exhibited the highest (~40 mm) and lowest (~3 mm) weekly cumulative rainfall values, respectively. Similarly, TS6 and TS4 were the hottest (~32 °C) and coldest (~24 °C) seasons, respectively. The TS6 season (April 15–June 14) with the highest ambient temperature (32.5 ± 1.3 °C) and relatively low weekly precipitation (12.4 ± 19.9 mm) was used as reference for modeling (Table 2).

**Figure 2:**
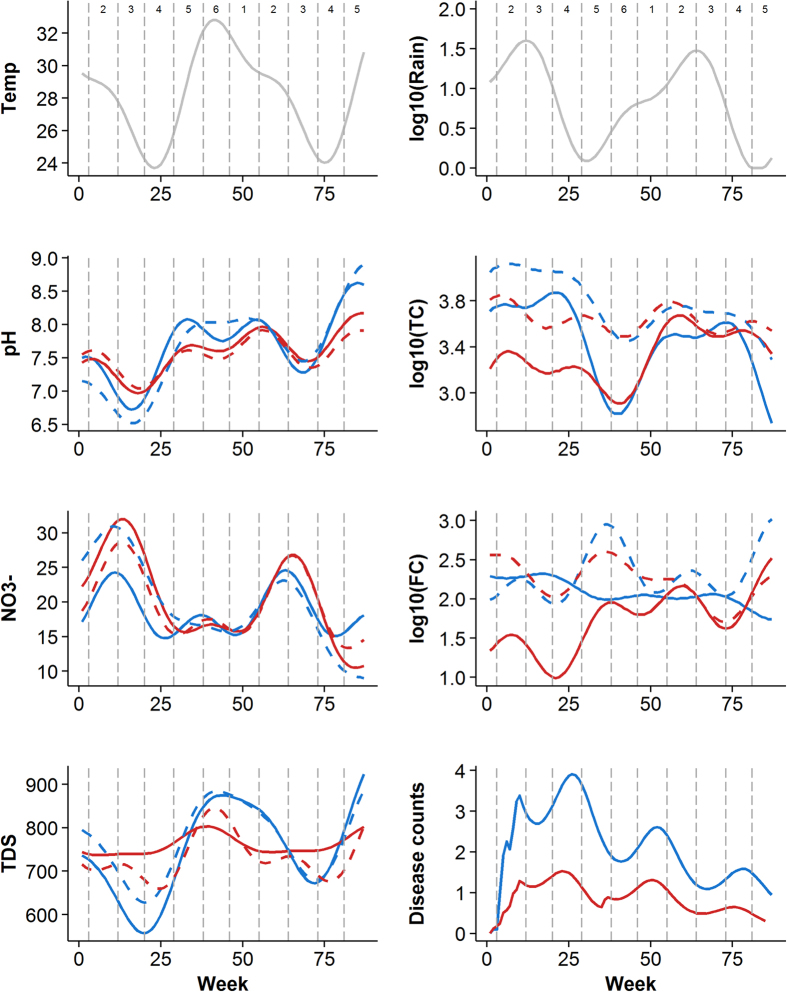
Fitted values of the double seasonal harmonic regression models (2A,B). Color represents study site (blue = urban; red = rural); line type represents sampling domain (solid = public tap; dashed = private household); vertical lines labeled 1 through 6 denote two-month long seasons according to the Tamil calendar: TS1–Jun to Aug, TS2–Aug to Oct, TS3–Oct to Dec, TS4–Dec to Feb, TS5–Feb to Apr, TS6–Apr to Jun.

WQ varied across the Tamil seasons. Consistent trends in pH were observed with similar seasonal patterns in public and private domains and urban and rural sites. Exploratory analysis suggested that pH may be lower in the seasons with the highest amount of rainfall and higher during hot and dry seasons (Table 2). Results of Model 1 A confirmed that in the private domain, pH for both urban and rural sites was significantly higher during the reference season as compared to other seasons (p < 0.01) (Supplemental Material, Table S3). Predicted high pH values based on the harmonic regression model also corresponded with higher temperatures (Fig. 2).

Seasonal patterns in NO_3_^−^ in both domains and both sites were similar and well pronounced (Fig. 2), with peak concentrations in seasons characterized by high amounts of rainfall (Table 2). For both sites in both domains NO_3_^−^ during the reference season (TS6) was 6.3–9.6 ppm (p < 0.001) lower than in TS2 (Supplemental Material, Table S2).

**Figure 3:**
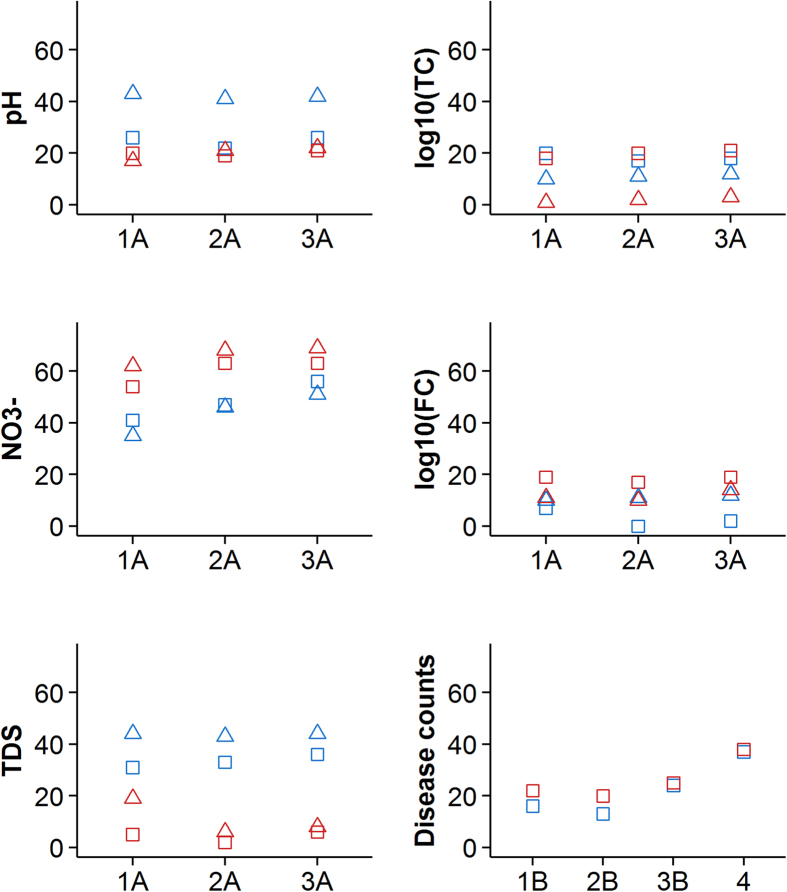
Q-values for the sequential WQ and diarrheal disease count models. Color represents study site (blue = urban; red = rural); shape represents sampling domain (square = public tap; triangle = private household). An increase in Q-value (Q = (null deviance–residual deviance)/null deviance*100%) indicates improved model fit with the additional parameters contributed by each sequential model.

Exploratory analysis suggested that TDS values peak during hot and dry seasons (Table 2; Supplemental Material, Table S2). The seasonal pattern in TDS was more pronounced in the urban than rural sites and in the private than public domain, as demonstrated by higher Q-values (Fig. 3). For both sites, significant reductions in private domain TDS values (16.3–25.8% in urban and 28.1–33.9% in rural, p < 0.001) were observed during seasons with substantial amounts of rainfall.

Seasonality in TC was more pronounced in the public domain in both study sites as compared to the private domain (Fig. 2). The highest public TC concentrations occurred during relatively wet seasons in both study sites with significantly higher values in TS2, TS3 and TS4 seasons as compared to the reference season (p < 0.001). In the rural sites, a 10-fold increase in weekly cumulative rainfall was associated with a 0.11 (CI_95%_: 0.02, 0.20) and 0.09 (CI_95%_: 0.00, 0.18) unit increase in public and private log_10_(TC) concentrations, respectively. However, in the urban sites, the relationship with rainfall was reversed, resulting in a reduction in TC concentration in both domains that was similar in magnitude (Supplemental Material, Table S2).

Seasonal fluctuations in FC concentrations were not well pronounced, particularly in the urban public domain. A relative peak in predicted values was observed in the private domain during high temperatures (TS5/TS6) in both urban and rural sites (Fig. 2). During the hottest season (TS6), private domain FC concentrations were significantly higher (up to one log_10_ in the rural sites p < 0.001) than in any other season (Supplemental Material, Table S2). However, after accounting for seasonality, in the private domain in the rural sites, with 1 °C increase in average temperature, log_10_(FC) concentration was likely to decrease by 0.13 (CI_95%_: 0.06, 0.21) units. In all other scenarios (urban public and private domains and rural public domain), this association with temperature was not significant (Supplemental Material, Table S2). Furthermore, model predicted values indicated that TC and FC concentrations in the private domain were higher than in the public domain throughout the study (Fig. 2).

### Temporal patterns in diarrheal disease counts

The disease count in the rural sites over the study period was approximately two times lower than in the urban sites (74 vs. 184 cases or 0.0015 vs. 0.0030 cases per person week of observation). A significant reduction in diarrheal disease counts of ~60% over the study period (RR = 0.41; CI_95%_: 0.28, 0.58) was observed in the urban sites and 75% (RR = 0.25; CI_95%_: 0.14, 0.44) in the rural sites, as estimated from Model 2 (Fig. 2). Two relative peaks in predicted diarrheal disease counts were observed, one in TS4 season, corresponding to lowest average temperature and lowest amount of rainfall and another in TS1 season characterized by relatively hot temperature and moderate rainfall (Fig. 2).

In the urban sites, the seasonal pattern of diarrheal disease counts was affected by temperature and precipitation, as indicated by a substantial improvement in model fit, or increase in Q-value (Fig. 3) from 13% to 24% with the addition of meteorological parameters. In the rural sites, the contribution of meteorological parameters was smaller yielding a 5% increase in Q-value (from 20% to 25%). After adjusting for overall trend, in the urban sites, weekly diarrheal disease counts decreased by 35% with each 1 °C increase in average temperature (RR = 0.65; CI_95%_: 0.55, 0.78); in the rural sites a similar statistically significant association was not observed. In the rural sites, diarrheal risk increased by 66% (RR = 1.66; CI_95%_: 1.11, 2.48) with a 10-fold increase in weekly cumulative rainfall relative to a minimal rainfall of 1 mm per week (Table 4); in the urban sites a similar association was not observed. Using the lagged rainfall variable in Model 3 resulted in statistically significant positive associations between rainfall and disease risk in urban (RR = 1.40; CI_95%_: 1.08, 1.80) and rural (RR = 1.82; CI_95%_: 1.22, 2.73) sites separately and for all sites combined (RR = 1.51; CI_95%_: 1.22, 1.88) (Table 4). Stronger association between rainfall and diarrheal risk in the rural than in the urban sites was maintained.

In the urban sites, after adjusting for trend, seasonality, and meteorological parameters, a 100-ppm increase in median TDS in the private domain doubled the diarrheal risk (RR = 2.23; CI_95%_: 1.12, 4.73). A substantial increase in diarrheal risk was also observed with a 10-fold increase in private domain TC concentration (RR = 4.25; CI_95%_: 1.24, 14.53). In contrast, a 10-fold increase in FC concentration was associated with a 68% reduction in diarrheal risk (RR = 0.32; CI_95%_: 0.18, 0.59) (Table 5, v.1). In the urban sites, these associations were maintained when using imputed private domain WQ values (Table 5, v.2). In the rural sites, no statistically significant associations between diarrheal disease counts and private domain WQ were observed.

## Discussion

Seasonality in the physicochemical WQ parameters in our study was well defined and seasonal peaks were consistent with other studies. Peak NO_3_^−^ concentration occurred during and immediately after a substantial amount of rainfall, as compared with dry conditions. This seasonal pattern is indicative of possible contaminant leaching from the soil (such as of nitrogen-containing fertilizers from agricultural applications)^10^. The lowest TDS levels were observed during and following periods of rainfall, potentially due to dilution. The seasonal patterns in NO_3_^−^ and TDS are consistent with the findings of Giridharan *et al*.^9^.

While heavy microbiological contamination was common across time, study sites, and domains, seasonal increases were observed. TC concentrations in the public domain in both study sites peaked during the wet seasons, with the lowest concentrations occurring in the hottest months. Given that the water systems are in poor structural condition, bacteria concentrations in piped water are expected to be influenced by infiltration of fecal contamination from the environment, which is more likely to occur during periods of heavy rainfall. Higher levels of bacterial contamination during wet conditions have been found in other studies in groundwater^8^ and surface water^30^ due to leaching and flushing effects. A recent meta-analysis reported that in most studies, peaks in indicator bacteria concentrations in drinking water occur during wet weather conditions. When WQ in piped systems specifically was considered, six studies conformed to this trend and two studies found higher contamination levels during dry weather conditions^31^.

At the same time that the lowest concentration of TC was observed (around week 40 of the study), particularly in the public domain, there was a peak in household FC concentration in both urban and rural sites. The positive differences in both TC and FC concentrations between public and private domains during most weeks of the study indicate that WQ is consistently lower in the private as compared to the public domain throughout the year. The magnitude of the difference is highest during times when concentrations in the public domain are lower. The phenomenon of an increase in bacterial contamination at the household level of cleaner source waters has been previously documented^32^. This difference is most pronounced around week 40 of the study, coinciding with the hottest season, which may be indicative of high indoor temperatures promoting bacterial growth in the storage containers. In the dry season, water availability is also lower, particularly in the urban sites, most likely necessitating longer storage times. Longer storage times allow for microbiological WQ changes due to natural growth and attenuation as well as anthropogenic causes (e.g. poor hygiene practices resulting from using insufficient quantities of water)^33^.

Controlling for trend and seasonality, rainfall was associated with an increase in public and private TC concentrations and in public FC concentrations in the rural sites. In the urban sites, no effect of rainfall on already high TC and FC concentrations in the public domain was observed, and both TC and FC concentrations decreased with increased rainfall in the private domain. A possible explanation for this dynamic is that in the rural sites, where outdoor contamination is high due to open defecation practices and animals being kept near the home^25^, wet weather conditions lead to higher bacteria concentrations in the water. In the urban sites, perhaps the predominating factor contributing to the reversed association with rainfall is the aforementioned seasonal water availability, meaning that lower amounts of rainfall lead to water scarcity, and hence lower microbiological WQ.

A strong downward trend, adjusted for population under observation, and moderate seasonal pattern were observed in the diarrheal disease counts. This trend predominated over the seasonality and was driven by the aging of the main contributors of reported cases (i.e. children under 5) and declining susceptibility to diarrhea over time. The overall higher diarrheal disease counts in the urban sites were most likely due to higher prevalence of overcrowding, an independent risk factor for diarrheal infections in the study area^25^. Two relative peaks in predicted diarrheal disease counts were observed, one in TS4 and another in TS1. The first peak coincided with cool and dry meteorological conditions, consistent with the seasonal pattern of rotavirus infections in tropical climates^13^,^15^, and specifically in Vellore^16^. The second peak coincided with warmer temperatures and the beginning of rainfall, which is closer to the seasonal pattern of *Cryptosporidium* and bacterial enteric infections^12^,^14^.

Apart from seasonality, independent effects of temperature and rainfall were also observed. In the urban sites, temperature decreased and rainfall (with 3-week lag) increased diarrheal risk. In the rural sites, rainfall also increased diarrheal risk and temperature exhibited no effect. The negative association with temperature found in our study is consistent with rotavirus seasonality studies^13^,^15^. The positive association with rainfall is consistent with most other studies examining this relationship; however, negative associations have also been found^17^. Contradicting findings among seasonality studies highlight the complex relationship between precipitation and temperature and the transmission of waterborne diseases and the need for examining how additional risk factors (e.g. geographical region, type of water supply, urban vs. rural setting) modify this relationship^17^. In our study, the reason behind rainfall being the predominant risk factor for diarrhea in the rural sites may be the aforementioned higher levels of outdoor fecal contamination with higher likelihood of exposure during wet conditions. In the urban sites, the effects of overcrowding on diarrhea may be exacerbated by high temperatures associated with lower water availability for hygiene and sanitation.

The detected associations between diarrheal disease counts and indicator bacteria concentrations while controlling for seasonality were inconsistent: we found a positive association with TC and negative association with FC in the urban sites. The lack of statistically significant associations in the rural sites is not surprising given the overall very high TC and FC concentrations in our study. Limited associations between indicator bacteria and diarrheal infections have been reported in other studies as well^20^,^21^. However, the statistically significant negative association between FC concentration and diarrheal disease counts in the urban sites is surprising. This finding could be an artifact of an imprecise WQ measurement due to high short-term variability in indicator bacteria^30^ characterizing which was not an objective of our study, opposing effects of hot and dry conditions on the health outcome and private domain FC in the urban sites, or the result of other co-occurring mediating factors to the relationship that were not taken into account in the analysis.

Waterborne disease epidemiology suffers from several limitations some of which also apply to our study. In terms of quantifying the exposure, the current microbiological WQ indicators exhibit high temporal variability^30^ and limited correlations with specific disease causing pathogens^18^. A limitation of our study in quantifying the exposure was the use of total and fecal coliforms to characterize microbiological WQ. A suggested improvement, following recent guidelines from the World Health Organization would be to use of *E. coli* due to its better performance as a pathogen presence indicator in water samples^19^, particularly in tropical regions^34^, and a stronger association with diarrheal risk^21^. Further development of cost-effective and easy to measure alternative indicators of fecal contamination of drinking water is still needed^20^,^35^,^36^,^37^, such as H_2_S^38^,^39^. Another limitation related to WQ collection in our study is the lack of replicate samples and multiple dilutions to confirm bacteria concentrations as a compromise for higher sampling frequency. In terms of quantifying the outcome, our study suffers from reliance on self-reporting rather than a more objective measure^21^ and underreporting due to a relatively long recall period of one week^25^,^40^.

With the noted limitations, the presented study utilized methodological advances in data collection (concurrent prospective disease surveillance and environmental monitoring) and analysis (preserving temporality within the data by using time series analysis), in order to quantify the effects of WQ on diarrheal risk^41^. Advantages offered by our analysis are the ability to distinguish the independent effects of meteorological parameters and WQ on diarrheal disease counts by modeling out trend and seasonality and addressing differences in the effects of these parameters in the urban vs. rural sites. As a result of a detailed analytical method, some contradictory findings arose, which deserve attention in future studies. Time series analysis is still relatively uncommon in waterborne disease epidemiology, as compared to air pollution studies^41^; the increased use of this approach in longitudinal studies will enable direct comparison of our findings to those of others.

Our study has also demonstrated the utility of a local calendar. This is not commonly done in seasonality studies but has been suggested in the literature, particularly in tropical regions where seasonal weather patterns are more subtle than in the temperate climates and may be driven by factors other than temperature and precipitation^15^. The Tamil calendar, based on the classical Hindu solar calendar, continues to be extensively used today for cultural, religious and agricultural events in the Tamil regions of south India. Comparable model fit using Tamil seasons and more complicated models with seasonal harmonics for all study parameters (Models 1 and 2 in Fig. 3) suggests the high potential of using the local calendar to frame risk communication and educational messaging in a way that is more relevant to people’s seasonal activities. It should be noted that direct translation of season names from Sanskrit to English lacks a perfect alignment with current predominant weather. For example, the reference season (April 15-June 14) “Vasanta” in Sanskrit, “ila-ventil” in English transliteration from Tamil, or “Light warmth” in English translation, is no longer the season with light warmth but more likely the period described as “Harsh warmth” due to changing climate patterns in Tamil Nadu^42^.

Our results demonstrated that improved water sources such as piped distribution systems, can have high seasonal fluctuations in WQ, associated with meteorological conditions. The findings support the notion that in order to quantify the extent of water contamination, sampling is necessary throughout the year. While contamination is more likely to be higher in the wet seasons^31^, variability in the seasonal patterns exists between water source types and geographic settings. Our findings also suggest that the study communities will benefit from targeted educational campaign on safe water storage practices, particularly during hot and dry seasons.

Diarrheal disease counts exhibited seasonal fluctuations along with age-related trends; future study designs should account for temporal variability in exposures and outcomes. Our study confirmed that associations among diarrheal disease counts, WQ and meteorological conditions and their seasonal trends can differ by urban vs. rural setting. Better understanding of the seasonal patterns in environmental exposures, health outcomes, and their links to local meteorological features is likely to guide in selecting the time window for interventions. Furthermore, improvements in routine WQ monitoring integrated with waterborne disease surveillance remain crucial in order to quantify and improve the impact of water infrastructure on health^43^.

## Additional Information

**How to cite this article**: Kulinkina, A. V. *et al*. Seasonality of water quality and diarrheal disease counts in urban and rural settings in south India. *Sci. Rep.* **6**, 20521; doi: 10.1038/srep20521 (2016).

## Supplementary Material

Supplementary Information

## Figures and Tables

**Table 1:** Study population, diarrheal disease counts and water quality (WQ) sampling.

Community	Houses	Population	Person-weeks of observation	Diarrheal disease count	Total taps	Sampled taps (%)	Public domain WQ samples	Private domain WQ samples
**Urban**	**160**	**852**	**61,977**	**184**	**61**	**36 (59)**	**357**	**654**
Kaspa (U1)	92	470	34,301	110	45	27 (60)	251	345
RNP (U2)	68	382	27,677	74	16	9 (56)	106	309
**Rural**	**140**	**727**	**47,423**	**74**	**117**	**64 (55)**	**705**	**564**
A. Kattupadi (R1)	39	189	14,661	29	25	13 (52)	151	165
Kattuputhur (R2)	41	232	16,617	35	61	33 (54)	391	168
K. Pudur (R3)	60	306	16,145	10	31	18 (58)	163	231
**Total**	**300**	**1,579**	**109,400**	**258**	**178**	**100 (56)**	**1,062**	**1,218**

**Table 2:** Study parameters (Mean ± SD) summarized by urban (U) and rural (R) study sites and Tamil calendar seasons.

		TS1 (Harsh warmth) Jun 15–Aug 14	TS2 (Dark clouds) Aug 15–Oct 14	TS3 (Chill, cold) Oct 15–Dec 14	TS4 (Early mist) Dec 15–Feb 14	TS5 (Late mist) Feb 15–Apr 14	TS6 (Light warmth) Apr 15–Jun 14
**Meteorological parameters**#							
Temperature (°C)	U/R	30.61 ± 1.17	28.92 ± 0.70	26.20 ± 1.43	24.23 ± 1.16	28.87 ± 1.82	32.51 ± 1.34
Rainfall (mm)	U/R	23.05 ± 28.35	33.23 ± 33.65	40.43 ± 27.74	7.37 ± 20.26	2.99 ± 11.84	12.44 ± 19.94
**Diarrheal episodes**							
Weekly counts	U	1.55 ± 1.37	2.22 ± 1.80	1.41 ± 1.62	2.94 ± 3.13	2.38 ± 2.99	1.88 ± 1.55
	R	0.73 ± 0.9	0.89 ± 0.96	0.65 ± 1.06	1.24 ± 1.3	0.57 ± 0.85	1.25 ± 1.04
**Public domain water quality**							
pH	U	7.92 ± 0.27	7.91 ± 0.45	7.25 ± 0.54	7.94 ± 0.57	7.98 ± 0.37	7.81 ± 0.47
	R	7.83 ± 0.39	7.83 ± 0.46	7.61 ± 0.45	7.46 ± 0.52	7.84 ± 0.37	7.53 ± 0.30
NO3- (ppm)	U	17 ± 3	24 ± 5	21 ± 4	16 ± 4	16 ± 2	17 ± 3
	R	15 ± 2	22 ± 4	27 ± 5	18 ± 5	15 ± 2	17 ± 2
TDS (ppm)	U	849 ± 134	805 ± 92	689 ± 84	715 ± 107	740 ± 83	884 ± 149
	R	713 ± 108	760 ± 108	741 ± 132	769 ± 133	744 ± 157	857 ± 312
TC (CFU/100mL)	U	5,536 ± 6,450	5,137 ± 4,540	5,989 ± 3,703	4,998 ± 3,786	4,257 ± 6,381	2,284 ± 4,305
	R	4,142 ± 4,464	5,673 ± 4,261	5,852 ± 3,733	4,020 ± 4,252	2,425 ± 3,104	2,200 ± 4,545
FC (CFU/100mL)	U	213 ± 189	264 ± 384	207 ± 167	162 ± 170	413 ± 661	390 ± 634
	R	153 ± 159	190 ± 166	205 ± 182	108 ± 231	207 ± 313	329 ± 493
**Private domain water quality**							
pH	U	7.99 ± 0.25	7.91 ± 0.74	7.38 ± 0.71	7.36 ± 0.60	7.86 ± 0.20	8.13 ± 0.31
	R	7.73 ± 0.39	7.77 ± 0.48	7.48 ± 0.52	7.32 ± 0.61	7.62 ± 0.39	7.56 ± 0.28
NO3- (ppm)	U	17 ± 2	23 ± 3	20 ± 5	19 ± 5	16 ± 3	17 ± 2
	R	16 ± 2	25 ± 4	25 ± 4	19 ± 4	16 ± 2	17 ± 2
TDS (ppm)	U	867 ± 116	766 ± 101	713 ± 88	667 ± 81	836 ± 48	890 ± 155
	R	751 ± 218	681 ± 145	739 ± 118	677 ± 115	710 ± 174	1,006 ± 361
TC (CFU/100mL)	U	6,952 ± 7,345	6,445 ± 5,318	5,958 ± 3,582	8,594 ± 7,248	5,185 ± 7,203	4,584 ± 5,229
	R	7,377 ± 6,721	6,677 ± 6,126	5,023 ± 3,842	4,572 ± 4,451	4,638 ± 5,219	7,830 ± 8,287
FC (CFU/100mL)	U	197 ± 221	492 ± 710	209 ± 159	419 ± 675	715 ± 753	805 ± 892
	R	342 ± 531	408 ± 705	152 ± 157	287 ± 584	462 ± 667	949 ± 938

^#^Weekly average temperature and cumulative rainfall are presented.

**Table 3:** Regression model specifications.

Model	Equation	Model specifications
1A	*x*_*t*_ = *β*_*0*_ + *β*_*1*_*t* + *β*_*2*_*TS1* + *β*_*3*_*TS2* + *β*_*4*_*TS3* + *β*_*5*_*TS4* + *β*_*6*_*TS5* + *e*_*t*_	*x*_*t*_ is the un-aggregated value of WQ parameter which occurred in *t*-week; *y*_*t*_ is the cumulative diarrheal disease count for *t*-week.
1B	*y*_*t*_ = *exp*{*β*_*0*_ + *β*_*1*_*t* + *β*_*2*_*TS1* + *β*_*3*_*TS2* + *β*_*4*_*TS3* + *β*_*5*_*TS4* + *β*_*6*_*TS5* + *e*_*t*_}	TS1 through TS5 are binary variables for Tamil season (Table 2); *β*_*1*_ regression coefficient reflects the trend over the study period; *β*_*2*_ through *β*_*6*_ reflect the change in the study parameter as compared to the season-specific reference category (TS6).
2A	*x*_*t*_ = *β*_*0*_ + *β*_*1*_*t* + *β*_*L*_*S* + *e*_*t*_	*x*_*t*_ is the un-aggregated value of WQ parameter which occurred in *t*- week; *y*_*t*_ is the cumulative diarrheal disease count for *t*-week.
2B	*y*_*t*_ = *exp*{*β*_*0*_ + *β*_*1*_*t* + *β*_*L*_*S* + *e*_*t*_}	*β*_*1*_ regression coefficient reflects the trend and *β*_*L*_ (*β*_*2*_ through *β*_*5*_) is the vector of coefficients for a seasonal pattern (S) represented by two harmonics^#^; a period ω = 365.25 is used to adjust for the effect of a leap year.
3A	*x*_*t*_ = *β*_*0*_ + *β*_*1*_*t* + *β*_*L*_*S* + *β*_*6*_ *Temp* + *β*_*7*_*Rain* + *e*_*t*_	*x*_*t*_ is the value of un-aggregated WQ parameter which occurred in *t*-week; *y*_*t*_ is the cumulative diarrheal disease count for *t*-week.
3B	*y*_*t*_ = *exp*{*β*_*0*_ + *β*_*1*_*t* + *β*_*L*_*S* + *β*_*6*_ *Temp* + *β*_*7*_*Rain* + *e*_*t*_}	Interpretation of *β*_*1*_ and *β*_*L*_is similar to Model 2; *β*_*6*_ and *β*_*7*_are effects of weekly average temperature and weekly cumulative rainfall (log_10_ transformed).
4	*y*_*t*_ = *exp*{*β*_*0*_ + *β*_*1*_*t* + *β*_*L*_*S* + *β*_*6*_ *Temp* + *β*_*7*_*Rain* + *β*_*8*_*pH* + *β*_*9*_*NO*_*3*_^−^ + *β*_*10*_*TDS* + *β*_*11*_*TC* + *β*_*12*_*FC* + *e*_*t*_}	*y*_*t*_ is the cumulative diarrheal disease count for *t*-week.
		Interpretation of *β*_*1*_, *β*_*L*_, *β*_*6*_ and *β*_*7*_are similar to Model 3; *β*_*8*_ through *β*_*12*_are effects of private domain weekly median water quality parameters (TC and FC were log_10_ transformed).

^#^*β*_*L*_ is equivalent to *β*_*2*_ sin (*2πωt*) + *β*_*3*_ cos (*2πωt*) + *β*_*4*_ sin (*4πωt*) + *β*_*5*_ cos (*4πωt*).

**Table 4:** Results of Model 3B–Relative risk of diarrhea [RR (95% CI)] associated with 1 °C increase in weekly average temperature and 1-log_10_ increase in weekly cumulative rainfall, adjusted for population under observation, trend and seasonality.

	Urban	Rural	Combined
**No temporal lags**	**[24%]**	**[25%]**	**[19%]**
Temperature	0.65 (0.55, 0.78)***	1.04 (0.79, 1.37)	0.75 (0.65, 0.87)***
Log_10_ (Rain)	0.74 (0.55, 1.01)	1.66 (1.11, 2.48)*	0.97 (0.76, 1.24)
**3-week lag for rain**	**[24%]**	**[27%]**	**[22%]**
Temperature	0.71 (0.60, 0.84)***	1.00 (0.76, 1.31)	0.78 (0.68, 0.90)***
Log_10_ (Rain)	1.40 (1.08, 1.80)*	1.82 (1.22, 2.73)**	1.51 (1.22, 1.88)***

Statistical significance is indicated by ***p < 0.001, **p < 0.01, *p < 0.05; Q-value of the model is denoted in brackets.

**Table 5:** Results of Model 4–Relative risk of diarrhea [RR (95% CI)] associated with median weekly water quality (WQ) parameters in the private domain, adjusted for population under observation, trend, seasonality, temperature and rainfall with no temporal lags.

	Urban	Rural	Combined
**v. 1 Raw WQ**	**[37%]**	**[38%]**	**[18%]**
Med pH	0.77 (0.35, 1.71)	0.72 (0.19, 2.68)	1.20 (0.72, 1.99)
Med NO_3_^−^	0.99 (0.91, 1.08)	1.10 (0.95, 1.28)	0.97 (0.92, 1.03)
Med TDS#	2.23 (1.12, 4.73)*	1.18 (0.91, 1.52)	1.15 (0.95, 1.40)
Med log_10_ (TC)	4.25 (1.24, 14.53)*	0.33 (0.08, 1.27)	1.50 (0.70, 3.25)
Med log_10_ (FC)	0.32 (0.18, 0.59)***	0.99 (0.49, 2.01)	0.74 (0.52, 1.04)
**v. 2 Imputed WQ**	**[30%]**	**[36%]**	**[18%]**
Med pH	1.03 (0.52, 2.05)	0.64 (0.18, 2.25)	1.42 (0.89, 2.26)
Med NO_3_^−^	0.95 (0.89, 1.01)	1.13 (0.98, 1.31)	0.96 (0.91, 1.01)
Med TDS^#^	1.53 (0.94, 2.50)	1.17 (0.95, 1.45)	1.14 (0.96, 1.34)
Med log_10_ (TC)	3.74 (1.45, 9.63)**	0.21 (0.07, 0.67)**	1.18 (0.62, 2.25)
Med log_10_ (FC)	0.41 (0.25, 0.67)***	1.11 (0.60, 2.04)	0.81 (0.59, 1.12)

^#^For each parameter, the relative risk associated with a 1 unit increase is presented with the exception of TDS (100 ppm is used). Statistical significance is indicated by ***p < 0.001, **p < 0.01, *p < 0.05; Q-value of the model is denoted in brackets.
