# Supplementary material for: Seasonality of water quality and diarrheal disease counts in urban and rural settings in south India
Source: Sci Rep. 2016 Feb 12;6:20521. doi: 10.1038/srep20521 (PMC4751522; doi:10.1038/srep20521)
Supplement: Supplementary Information [file srep20521-s1.pdf]

SUPPLEMENTAL MATERIAL

Seasonality of water quality and diarrheal disease counts in urban and rural settings in south India

Alexandra V. Kulinkina, Venkat Raghava Mohan, Mark R. Francis, Deepthi Kattula, Rajiv Sarkar, Jeanine D. Plummer, Honorine Ward, Gagandeep Kang, Vinohar Balraj, Elena N. Naumova

Table S1: Spearman's correlation coefficients for weekly aggregated meteorological and private domain water quality parameters used as predictors in the diarrheal disease count models. Urban site correlations are shown in the top and rural site on the bottom portions of the matrix.

	Temp	Rain (log_e)	pH	NO_3^-	TDS	TC (log_{10})	FC (log_{10})
Temp	---	0.04	0.48*	-0.20	0.80*	-0.49*	0.40*
Rain (log_e)	0.07	---	0.13	0.30	0.24	-0.25	-0.25
pH	0.33*	-0.07	---	-0.05	0.68*	-0.30	0.27
NO_3^-	-0.34	0.18	-0.19	---	-0.12	0.37*	0.07
TDS	0.02	0.05	0.15	-0.28	---	-0.47*	0.39*
TC (log_{10})	0.00	0.18	0.07	0.14	0.14	---	0.18
FC (log_{10})	0.44*	-0.09	0.05	-0.16	-0.02	0.20	---

* Indicates statistical significance at $\alpha < 0.05$ level

Table S2: Results of water quality regression models 1A, 2A and 3A

	pH		NO_3^- (ppm)		TDS (ppm)		TC (log_{10})<br>(CFU/100 mL)		FC (log_{10})<br>(CFU/100 mL)	
	Urban	Rural	Urban	Rural	Urban	Rural	Urban	Rural	Urban	Rural
**MODEL 1A#**										
**PUBLIC**	[26]	[20]	[41]	[54]	[31]	[5]	[20]	[18]	[7]	[19]
Intercept	8.727***<br>(0.429)	7.178***<br>(0.064)	17.14***<br>(3.295)	19.67***<br>(0.532)	901.2***<br>(104.6)	864.9***<br>(24.62)	5.993***<br>(0.569)	2.780***<br>(0.079)	5.128***<br>(0.674)	1.589***<br>(0.099)
Week	-0.022*<br>(0.010)	0.008***<br>(0.001)	0.004<br>(0.077)	-0.068***<br>(0.009)	-0.392<br>(2.430)	-0.191<br>(0.414)	-0.071***<br>(0.013)	0.006***<br>(0.001)	-0.071***<br>(0.016)	0.010***<br>(0.002)
TS1	0.305*<br>(0.122)	0.236***<br>(0.064)	-0.584<br>(0.940)	-0.933<br>(0.535)	-31.79<br>(29.85)	-142.2***<br>(24.77)	1.091***<br>(0.162)	0.196*<br>(0.079)	0.572**<br>(0.190)	-0.309**<br>(0.099)
TS2	0.468*<br>(0.191)	0.166**<br>(0.060)	6.905***<br>(1.465)	6.343***<br>(0.498)	-72.65<br>(46.52)	-93.80***<br>(23.10)	1.701***<br>(0.253)	0.495***<br>(0.074)	1.051***<br>(0.297)	-0.132<br>(0.093)
TS3	-0.026<br>(0.259)	-0.130<br>(0.072)	3.617<br>(1.990)	11.57***<br>(0.601)	-185.7**<br>(63.18)	-110.8***<br>(27.83)	2.443***<br>(0.344)	0.479***<br>(0.089)	1.762***<br>(0.404)	-0.196<br>(0.111)
TS4	0.846*<br>(0.339)	-0.169**<br>(0.057)	-1.390<br>(2.606)	1.882***<br>(0.472)	-156.9<br>(82.74)	-85.64***<br>(21.85)	2.887***<br>(0.450)	0.292***<br>(0.070)	2.129***<br>(0.530)	-0.737***<br>(0.088)
TS5	-0.047<br>(0.127)	0.326***<br>(0.061)	-1.279<br>(0.975)	-1.593**<br>(0.504)	-148.6***<br>(30.97)	-113.7***<br>(23.34)	-0.536**<br>(0.168)	0.086<br>(0.075)	-0.918***<br>(0.200)	-0.146<br>(0.094)
**PRIVATE**	[43]	[17]	[35]	[62]	[44]	[19]	[10]	[1]	[10]	[11]
Intercept	7.321***<br>(0.075)	7.269***<br>(0.082)	22.06***<br>(0.554)	19.86***<br>(0.546)	853.3***<br>(15.94)	987.6***<br>(31.97)	3.819***<br>(0.086)	3.675***<br>(0.096)	2.739***<br>(0.127)	3.045***<br>(0.144)
Week	0.019***<br>(0.001)	0.0073***<br>(0.001)	-0.129***<br>(0.010)	-0.070***<br>(0.007)	0.864**<br>(0.290)	0.469<br>(0.434)	-0.007***<br>(0.002)	-0.005<br>(0.001)	0.000<br>(0.002)	-0.004*<br>(0.002)
TS1	-0.323***<br>(0.071)	0.0971<br>(0.083)	1.707**<br>(0.520)	-0.341<br>(0.552)	-31.58*<br>(14.95)	-260.0***<br>(32.27)	0.179*<br>(0.081)	0.018<br>(0.097)	-0.747***<br>(0.119)	-0.646***<br>(0.145)
TS2	-0.558***<br>(0.075)	0.0536<br>(0.095)	8.212***<br>(0.553)	9.641***<br>(0.631)	-139.0***<br>(15.91)	-335.0***<br>(36.92)	0.353***<br>(0.086)	0.059<br>(0.111)	-0.368**<br>(0.127)	-0.553***<br>(0.166)
TS3	-1.243***<br>(0.076)	-0.2484**<br>(0.088)	6.731***<br>(0.555)	9.471***<br>(0.581)	-199.0***<br>(15.95)	-278.1***<br>(34.00)	0.351***<br>(0.087)	-0.075<br>(0.103)	-0.602***<br>(0.127)	-0.979***<br>(0.153)
TS4	-0.721***<br>(0.058)	-0.3257***<br>(0.080)	1.739***<br>(0.427)	3.158***<br>(0.529)	-220.5***<br>(12.28)	-334.7***<br>(30.95)	0.385***<br>(0.067)	-0.096<br>(0.093)	-0.674***<br>(0.098)	-0.835***<br>(0.139)
TS5	-0.149<br>(0.083)	0.0352<br>(0.084)	-1.045<br>(0.613)	-1.122*<br>(0.559)	-48.84**<br>(17.61)	-298.2***<br>(32.73)	-0.053<br>(0.096)	-0.144<br>(0.099)	-0.084<br>(0.140)	-0.527***<br>(0.147)
**MODEL 2A**										
**PUBLIC**	[22]	[19]	[47]	[63]	[33]	[2]	[17]	[20]	[0]	[17]
Intercept	7.242***<br>(0.186)	7.191***<br>(0.061)	18.13***<br>(1.323)	24.22***<br>(0.449)	664.6***<br>(43.64)	758.0***<br>(23.75)	3.596***<br>(0.246)	3.021***<br>(0.074)	2.289***<br>(0.298)	1.215***<br>(0.094)
Week	0.011**<br>(0.004)	0.009***<br>(0.001)	0.006<br>(0.025)	-0.099***<br>(0.008)	2.217**<br>(0.819)	0.133<br>(0.439)	-0.005<br>(0.005)	0.006***<br>(0.001)	-0.005<br>(0.006)	0.012***<br>(0.002)
Sin (2π)	-0.382***<br>(0.068)	-0.135***<br>(0.028)	3.042***<br>(0.487)	6.389***<br>(0.209)	-92.22***<br>(16.05)	-30.12**<br>(11.05)	0.401***<br>(0.091)	0.240***<br>(0.034)	0.087<br>(0.109)	-0.138**<br>(0.044)
Cos (2π)	0.081*<br>(0.036)	0.151***<br>(0.022)	0.887***<br>(0.257)	0.126<br>(0.162)	90.61***<br>(8.475)	-0.687<br>(8.544)	-0.099*<br>(0.048)	0.040<br>(0.026)	-0.009<br>(0.057)	0.204***<br>(0.034)
Sin (4π)	0.275***<br>(0.048)	0.185***<br>(0.024)	1.292***<br>(0.344)	-0.769***<br>(0.177)	21.31<br>(11.36)	-0.156<br>(9.359)	-0.012<br>(0.064)	0.104***<br>(0.029)	-0.047<br>(0.076)	0.196***<br>(0.037)
Cos (4π)	0.157***<br>(0.036)	0.054*<br>(0.022)	-2.630***<br>(0.257)	-2.674***<br>(0.166)	-14.85<br>(8.476)	-10.03<br>(8.761)	0.179***<br>(0.048)	0.091***<br>(0.027)	0.012<br>(0.058)	-0.125***<br>(0.035)

**PRIVATE**	[41]	[21]	[46]	[68]	[43]	[6]	[11]	[2]	[11]	[10]
Intercept	6.899***<br>(0.070)	7.289***<br>(0.060)	25.85***<br>(0.460)	21.02***<br>(0.372)	739.0***<br>(14.53)	728.3***<br>(25.48)	4.041***<br>(0.078)	3.669***<br>(0.071)	2.266***<br>(0.114)	2.515***<br>(0.107)
Week	0.018***<br>(0.001)	0.006***<br>(0.001)	-0.150***<br>(0.009)	-0.038***<br>(0.007)	0.995***<br>(0.289)	0.335<br>(0.467)	-0.007***<br>(0.002)	-0.001<br>(0.001)	0.002<br>(0.002)	-0.006**<br>(0.002)
Sin (2π)	-0.482***<br>(0.036)	-0.070*<br>(0.032)	5.071***<br>(0.241)	5.099***<br>(0.198)	-82.40***<br>(7.598)	-55.73***<br>(13.55)	0.196***<br>(0.041)	0.059<br>(0.034)	-0.319***<br>(0.060)	-0.217***<br>(0.057)
Cos (2π)	0.201***<br>(0.030)	0.161***<br>(0.028)	1.062***<br>(0.201)	0.202<br>(0.174)	76.56***<br>(6.339)	35.71**<br>(11.90)	-0.081*<br>(0.034)	0.055<br>(0.033)	-0.058<br>(0.050)	0.109*<br>(0.050)
Sin (4π)	0.167***<br>(0.035)	0.192***<br>(0.031)	0.431<br>(0.229)	-0.396*<br>(0.196)	17.92*<br>(7.228)	-13.21<br>(13.41)	0.044<br>(0.039)	0.086*<br>(0.038)	0.172**<br>(0.057)	0.139*<br>(0.056)
Cos (4π)	0.050<br>(0.027)	0.058<br>(0.030)	-1.506***<br>(0.177)	-3.055***<br>(0.189)	-15.49**<br>(5.594)	-39.53**<br>(12.92)	0.067*<br>(0.030)	0.065<br>(0.036)	-0.233***<br>(0.044)	-0.068<br>(0.054)
**MODEL 3A**										
**PUBLIC**	[26]	[21]	[56]	[63]	[36]	[6]	[18]	[21]	[2]	[19]
Intercept	10.44***<br>(0.904)	9.219***<br>(0.504)	-15.69**<br>(5.994)	24.42***<br>(3.765)	190.8<br>(211.2)	1819***<br>(194.6)	4.550***<br>(1.218)	3.587***<br>(0.613)	-0.008<br>(1.474)	2.249**<br>(0.780)
Week	0.008*<br>(0.004)	0.009***<br>(0.001)	0.040<br>(0.023)	-0.101***<br>(0.008)	2.721**<br>(0.825)	0.155<br>(0.437)	-0.005<br>(0.005)	0.006***<br>(0.001)	-0.003<br>(0.006)	0.011***<br>(0.002)
Sin (2π)	-0.623***<br>(0.094)	-0.309***<br>(0.049)	3.844***<br>(0.625)	6.214***<br>(0.369)	-89.71***<br>(22.00)	-123.5***<br>(19.07)	0.372**<br>(0.127)	0.150*<br>(0.060)	0.345*<br>(0.155)	-0.289***<br>(0.077)
Cos (2π)	0.423***<br>(0.101)	0.362***<br>(0.061)	-2.737***<br>(0.670)	-0.001<br>(0.458)	39.76<br>(23.60)	107.6***<br>(23.65)	0.004<br>(0.136)	0.060<br>(0.075)	-0.252<br>(0.165)	0.254**<br>(0.095)
Sin (4π)	0.265***<br>(0.048)	0.205***<br>(0.025)	1.415***<br>(0.316)	-0.684***<br>(0.185)	23.12*<br>(11.14)	11.31<br>(9.555)	-0.016<br>(0.064)	0.131***<br>(0.030)	-0.044<br>(0.076)	0.239***<br>(0.038)
Cos (4π)	0.009<br>(0.053)	-0.035<br>(0.039)	-1.299***<br>(0.353)	-2.589***<br>(0.252)	2.655<br>(12.45)	-54.90***<br>(13.04)	0.140<br>(0.072)	0.091*<br>(0.041)	0.132<br>(0.088)	-0.133*<br>(0.052)
Temperature	-0.108***<br>(0.029)	-0.071***<br>(0.017)	1.074***<br>(0.195)	-0.014<br>(0.130)	14.73*<br>(6.877)	-37.28***<br>(6.694)	-0.031<br>(0.040)	-0.022<br>(0.021)	0.080<br>(0.048)	-0.039<br>(0.027)
Rain ($\log_{10}$)	0.035<br>(0.042)	-0.003<br>(0.036)	1.823***<br>(0.285)	0.419<br>(0.268)	36.53***<br>(10.04)	4.280<br>(13.88)	-0.043<br>(0.058)	0.110*<br>(0.044)	-0.126<br>(0.071)	0.163**<br>(0.055)
**PRIVATE**	[42]	[22]	[51]	[69]	[44]	[8]	[12]	[3]	[12]	[14]
Intercept	9.494***<br>(0.810)	8.107***<br>(0.597)	2.636<br>(5.137)	16.90***<br>(3.675)	537.4**<br>(169.2)	1189***<br>(253.9)	2.153*<br>(0.913)	4.590***<br>(0.715)	-0.493<br>(1.336)	6.492***<br>(1.057)
Week	0.017***<br>(0.002)	0.006***<br>(0.001)	-0.159***<br>(0.010)	-0.041***<br>(0.007)	0.774*<br>(0.323)	0.570<br>(0.468)	-0.005**<br>(0.002)	-0.001<br>(0.001)	0.005*<br>(0.003)	-0.006**<br>(0.002)
Sin (2π)	-0.667***<br>(0.078)	-0.157**<br>(0.059)	5.840***<br>(0.493)	5.679***<br>(0.364)	-82.52***<br>(16.23)	-105.5***<br>(25.12)	0.402***<br>(0.088)	-0.041<br>(0.071)	-0.016<br>(0.128)	-0.494***<br>(0.105)
Cos (2π)	0.490***<br>(0.092)	0.209**<br>(0.069)	-2.074***<br>(0.582)	0.217<br>(0.424)	45.07*<br>(19.18)	61.75*<br>(29.25)	-0.247*<br>(0.104)	0.105<br>(0.082)	-0.299*<br>(0.152)	0.614***<br>(0.122)
Sin (4π)	0.172***<br>(0.035)	0.229***<br>(0.034)	0.261<br>(0.219)	-0.784***<br>(0.210)	15.48*<br>(7.210)	8.065<br>(14.47)	0.051<br>(0.039)	0.129**<br>(0.041)	0.182**<br>(0.057)	0.099<br>(0.060)
Cos (4π)	-0.074<br>(0.046)	0.045<br>(0.044)	-0.234<br>(0.291)	-3.196***<br>(0.274)	-3.183<br>(9.597)	-45.93*<br>(18.91)	0.144**<br>(0.052)	0.053<br>(0.053)	-0.121*<br>(0.076)	-0.348***<br>(0.079)
Temperature	-0.087**<br>(0.027)	-0.031<br>(0.021)	0.775***<br>(0.173)	0.176<br>(0.128)	6.671<br>(5.696)	-17.68*<br>(8.854)	0.064*<br>(0.031)	-0.035<br>(0.025)	0.094*<br>(0.045)	-0.132***<br>(0.037)
Rain ($\log_{10}$)	-0.035<br>(0.042)	0.077*<br>(0.039)	1.839***<br>(0.266)	-1.009***<br>(0.242)	27.82**<br>(8.756)	45.06**<br>(16.70)	-0.099*<br>(0.047)	0.092*<br>(0.047)	-0.150*<br>(0.069)	-0.296***<br>(0.069)

Reference category for Model 1A is TS6 (spring).

Regression coefficient (Standard Error) are presented; statistical significance is indicated by ***p < 0.001, **p < 0.01, *p < 0.05. Q-value of the model denoted in brackets.

Table S3: Results of diarrheal disease count regression models 1B, 2B, 3B and 4 with no temporal lags

	Urban	Rural	Combined
**MODEL 1B**	[16]	[22]	[18]
Intercept	-5.443*** (0.294)	-5.336*** (0.390)	-5.414*** (0.233)
Week	-0.016*** (0.003)	-0.025*** (0.006)	-0.020*** (0.003)
TS1	0.141 (0.355)	-0.139 (0.476)	0.042 (0.284)
TS2	0.327 (0.305)	0.092 (0.405)	0.259 (0.242)
TS3	-0.345 (0.331)	-0.426 (0.439)	-0.361 (0.263)
TS4	0.480 (0.295)	0.390 (0.384)	0.467* (0.233)
TS5	0.382 (0.305)	-0.352 (0.474)	0.236 (0.249)
**MODEL 2B**	[13]	[20]	[15]
Intercept	-5.196*** (0.159)	-5.293*** (0.257)	-5.219*** (0.135)
Week	-0.017*** (0.003)	-0.027*** (0.006)	-0.021*** (0.003)
Sin (2π)	-0.009 (0.118)	0.083 (0.183)	0.016 (0.098)
Cos (2π)	0.023 (0.106)	0.022 (0.164)	0.003 (0.089)
Sin (4π)	0.077 (0.106)	-0.060 (0.168)	0.050 (0.089)
Cos (4π)	0.284* (0.111)	0.334 (0.173)	0.298** (0.093)
**MODEL 3B**	[24]	[25]	[19]
Intercept	7.136** (2.509)	-6.786 (4.063)	2.984 (2.103)
Week	-0.017*** (0.003)	-0.026*** (0.006)	-0.020*** (0.003)
Sin (2π)	-0.874*** (0.232)	-0.080 (0.380)	-0.655*** (0.197)
Cos (2π)	1.375*** (0.299)	-0.276 (0.451)	0.846*** (0.246)
Sin (4π)	0.162 (0.110)	-0.016 (0.173)	0.123 (0.093)
Cos (4π)	-0.399* (0.178)	0.500 (0.268)	-0.118 (0.146)
Temperature	-0.425*** (0.087)	0.035 (0.141)	-0.288*** (0.073)
Rain (log_{10})	-0.295 (0.156)	0.507* (0.204)	-0.027 (0.123)
**MODEL 4#**	[37]	[38]	[18]
Intercept	-11.84 (6.313)	-2.993 (8.552)	-3.240 (4.218)
Week	0.023 (0.018)	-0.016 (0.011)	-0.011 (0.007)
Sin (2π)	-0.052 (0.569)	-0.237 (0.794)	-0.057 (0.385)
Cos (2π)	-0.359 (0.632)	-0.539 (0.910)	0.300 (0.422)
Sin (4π)	0.045 (0.237)	0.254 (0.354)	-0.170 (0.173)
Cos (4π)	-0.441 (0.301)	0.890 (0.554)	-0.396 (0.242)
Temperature	-0.068 (0.169)	0.007 (0.229)	-0.180 (0.120)
Rain (log_{10})	-0.558* (0.272)	0.542 (0.347)	-0.137 (0.168)
Med pH	-0.261 (0.407)	-0.332 (0.672)	0.182 (0.259)
Med NO_3^-	-0.009 (0.042)	0.098 (0.074)	-0.026 (0.028)
Med TDS	0.008* (0.004)	0.002 (0.001)	0.001 (0.001)
Med TC (log_{10})	1.447* (0.627)	-1.120 (0.695)	0.407 (0.393)
Med FC (log_{10})	-1.127*** (0.307)	-0.012 (0.362)	-0.306 (0.174)

Results of version 1 are presented, where only private domain weekly median values are used with no interpolation.

Regression coefficient (Standard Error) are presented; statistical significance is indicated by ***$p < 0.001$, **$p < 0.01$, *$p < 0.05$. Q-value of the model is denoted in brackets.
